# Measurement of the Prescription of Physical Exercise in Chronic Diseases: A Proposal of a Scale for Physicians of Second-Level Hospital Practitioners

**DOI:** 10.3390/healthcare13232997

**Published:** 2025-11-21

**Authors:** Jesús Mauro Hernández Ramírez, Blanca R. Rangel_Colmenero, Eduardo Barrera_Juárez, Minerva Vanegas_Farfano, Daniel Carranza_Bautista, Ricardo López_García, Gloria Leticia Corrales Félix

**Affiliations:** 1 Facultad de Organización Deportiva, Universidad Autónoma de Nuevo León, Av. Universidad s/n, Ciudad Universitaria, San Nicolas de los Garza 66450, Mexico; jesus.hernandezrmz@uanl.edu.mx (J.M.H.R.); minerva.vanegasfr@uanl.edu.mx (M.V.);; 2 Escuela Nacional de Medicina, Instituto Tecnológico y de Estudios Superiores De Monterrey, Av. Eugenio Garza Sada 2501 Sur, Monterrey 64849, Mexico; eduardo.barrera@tec.mx

**Keywords:** physical activity, exercise prescription, chronic non-communicable diseases, hospital care, clinical practice

## Abstract

**Background:** Physical inactivity is one of the main risk factors for the development of chronic non-communicable diseases. Despite global initiatives such as “Exercise is Medicine”, there is little information regarding the recommendations of physicians in secondary public hospitals to implement physical activity as an adjuvant for their patients with CNDs. There is a critical gap between this knowledge and its implementation in routine clinical practice. This gap is particularly relevant in the context of a secondary hospital in Mexico, where CNDs are the leading cause of death. **Objectives:** The aim was to explore the perception and practice of physicians at a secondary care hospital in Nuevo León, Mexico, in relation to exercise prescription for the treatment of CNDs. **Methods**: Instrumental design research was conducted for the construction and valuation of the psychometric properties of a questionnaire administered to 127 physicians from a local hospital. AFE, CFA, and reliability analyses were conducted. **Results**: The analysis showed a two-factor structure in a scale with good reliability and adjustment according to the CFA goodness-of-fit indices. Also, it is shown that although 63.8% of physicians frequently inquire about PA in their patients and 60.6% discuss its importance, only 44.9% perform formal evaluations through physical tests. **Conclusions**: Barriers identified include a lack of standardized protocols, insufficient resources, and limitations in medical training. These findings highlight the need for institutional policies that prioritize exercise prescription as an essential component of CDN treatment, aligning with WHO guidelines for exploring population health.

## 1. Introduction

Physical inactivity is one of the main risk factors for mortality from non-treatable diseases, so people with insufficient levels of physical activity have a 20% to 30% higher risk of death compared to people who achieve a sufficient level of physical activity [1].

It has been proven that regular physical activity helps to prevent and treat chronic non-communicable diseases (CNDs) such as heart disease, stroke, diabetes, and breast and colon cancer. Likewise, it also helps to prevent hypertension, overweight, and obesity and can improve mental health, quality of life, and well-being [2].

The increase in physical activity at the population level has become an essential component of the main world initiators to improve health [3].

More structured PA counseling uses established behavioral strategies to change the individual’s lifestyle behavior, which is why it is suggested that physical exercise should also be part of in-hospital services [4].

To support the efforts of healthcare professionals to increase physical activity levels among their outpatients, in initiative called Exercise is Medicine (EIM) was launched in 2007 in conjunction with the American College of Sports Medicine and the American Medical Association. It highlights three principles underlying the EIM initiative: first, physical activity must be monitored as a vital sign; second, physical activity is an effective physical activity modality and should be prescribed; and third, the success of this vision requires joint efforts among three key stakeholder groups: physicians, exercise professionals, and patients [5].

There are initiatives for the implementation of EIM in primary care, but it has been suggested that EIM should also be part of the hospital care system (secondary and tertiary) with regard to treatment and prescription [6]. However, the implementation of these initiatives in routine clinical practice faces numerous barriers, including lack of time during consultations and gaps in medical training on exercise prescription, particularly in health systems with limited resources [7].

Currently, there is interest in knowing the reality, future, and medical expectations regarding the inclusion of exercise prescription in the state of Nuevo León, Mexico. It is important to be aware of this in order to promote new structured projects with scientifically based exercise prescriptions to improve the healthcare system. This leads us to ask the following research question: Do physicians suggest physical exercise to their patients to treat chronic, non-communicable diseases?

In addition, do they (physicians) consider it necessary that their patients be prescribed physical exercise by qualified exercise professionals in a second-level hospital? Therefore, the objective of this study is to exploit the level of prescription or recommendation of physical exercise by physicians to treat non-communicable diseases in a second-level public hospital.

## 2. Materials and Methods

### 2.1. Study Design

This publication corresponds to an empirical and instrumental design study conducted following the research methodology of [8]. This method is appropriate for understanding medical considerations regarding the prescription of physical activity in a second-level hospital. The data collection period lasted for 2 months and included two phases. Phase one sought to gain feedback for refinement or elimination of items; expert reviewers were contacted. The aim of phase two was to explore the validity of the structure of the scale. A third analysis sought confirmatory data of the scale.

### 2.2. Data Source

The study population consisted of active physicians at the Metropolitan Hospital of Nuevo León, Mexico, totaling approximately 140 active physicians. Procedures for selecting participants were followed according to the phases’ aims: two independent participant samples were recruited for this study. Sample 1 includes participation in the experts’ preliminary items analysis. Sample 2 was used for the EFA and a first consistency reliability analysis. For the third stage, the CFA analysis, 134 samples were obtained via bootstrapping of Sample 2.

#### 2.2.1. Participant Inclusion and Exclusion Criteria

For both phases, physicians with voluntary participation, male or female, employed at the hospital in question, from any age, who diagnose and treat patients with NCDs were sought and included. Exclusion criteria were as follows: Physicians without voluntary participation were excluded. Additionally, participants who did not complete the survey were removed from the sample. Specific information on participant criteria is presented in the following sections.

##### Expert Preliminary Items Analysis Participants

The sampling method implemented was non-probability, purposive sampling [9]. The intended sample followed the suggestion of the methodology in [10] of using at least three to five judges or experts. For this manuscript, we convened 9 experts. Eligibility criteria included professional knowledge in physical activity and sports sciences or medical specialization in emergency, cardiology, or internal medicine.

##### EFA and Consistency Reliability Participants

A non-probability convenience sampling was implemented since only the data of those people available and willing to share their data participated [9]. The intended study sample was 100 or greater, following the suggestions of [11]. Participants in this study included 132 physicians, of which 7 were excluded for not meeting the participation criteria.

### 2.3. Instrument

The questionnaire designed by [12], was used as a basis, to which our study added a new item: “Do you think it is necessary for specialized professionals to prescribe exercise to aid in the prevention or treatment of NCDs?” This retained the 5-point Likert format (1: never to 5: always) used by [12]. It was translated from English to Spanish and from Spanish to English to maintain the intended meaning of each item, highlighting the need for linguistic and cultural equivalence between versions [13].

### 2.4. Research Procedure

#### Expert Preliminary Items Analysis

A translated questionnaire was evaluated based on the Delphi method validation criteria [14] to ensure its content validity. The process was guided by the following methodological principles: *Selection of Experts*. Participants were systematically chosen based on their experience and specialization in the study area. *Anonymity and Independence*. Responses were collected anonymously and without interaction among the experts, minimizing group influence bias. *Process Control.* The researcher supervised each stage to ensure compliance with the established protocol. *Interactive Feedback*. Two-way communication was facilitated between the researcher and the experts to clarify doubts during the evaluation rounds. The validation process was implemented through iterative evaluation rounds with a panel of nine experts (N = 9).

Experts rated each item of the scale on a 4-point Likert scale: none = 0, slight = 1, sufficient = 2, much = 3. This multi-center approach served a dual purpose: first, it strengthened the content validity by ensuring that the items comprehensively reflected the theoretical constructs, and second, it generated empirical evidence regarding their representativeness. Conclusions were drawn based on the consensus reached by the panel, supporting the validity of the instrument. Additionally, a comprehensive statistical analysis was implemented to quantify the level of agreement among the experts using the Delphi method. This systematic approach allowed for the assessment of the robustness of the scale across four critical dimensions, defined according to the validation criteria of [8] and adapted to the context of this research by [15]. The survey was transferred to a Google Forms platform, a QR code was generated, and an invitation was sent to all departments of medicine at the metropolitan hospital, asking them to scan the QR code with their own mobile phones and complete the survey, which was estimated to take approximately 8 min to complete.

### 2.5. Ethical Statement

A protocol was established in accordance with the guidelines set forth in the General Law of Health Research in Mexico (latest reforms, 2014, Mexico) and presented to the Research Ethics Committee of the Metropolitan Hospital of Nuevo León, obtaining official approval, HMBSSSNL 2024/1018, once all their ethical standards were met.

For phases one and two, to maintain the confidentiality of the participants, the questionnaire asked them to anonymously and voluntarily participate in an online survey, only optionally including their name in the survey. No payments were made to the participants in any phase.

### 2.6. Analytical Approach

#### 2.6.1. Expert Preliminary Item Analysis

The evaluation was conducted using a standardized rating scale that examined the following: *Conceptual Coherence*. This evaluates how each item reflects the theoretical constructs of the study, ensuring that there are no ambiguities and that there is alignment with the research objectives to guarantee that the data is relevant and capable of answering the questions or hypotheses posed. *Linguistic Validity*. This ensures that the items are uniformly understood by the participants, including syntactic and semantic analysis, clarity assessment to eliminate ambiguities, and consideration of the educational level and sociocultural context of the population. *Relevance as an Indicator*. Its formulation must accurately capture the construct and provide analytical value. *Methodological Importance*. It should be essential for covering the dimensions of the construct and generating actionable data that can test hypotheses, thereby avoiding measurement gaps. Inclusion is justified through the previous literature and coherence with the conceptual framework.

To determine the validity index of the experts [16], recommendation was followed by using the total content validity coefficient (CVCt) method [10]. This method helps assess the degree of agreement among the experts. The author of this methodology suggests the participation of at least three to five judges or experts; for our validation, we convened nine experts.

Equation (1): this is the equation of the total content validity coefficient (CVCt). Note: this was extracted from Hernández-Nieto (2002, p. 72) [10].

(1) CVCt=∑CvctcN=∑[[∑XiJVmx]−Pei]1N

#### 2.6.2. EFA, CFA, and Reliability Analysis

The data analysis was based on descriptive statistics, conducted through a quantitative analysis using a data matrix analyzed via the statistical programs Microsoft^®^ Excel^®^ for Microsoft 365 MSO (vertion 2509 compilation 16.0.19231.20138) of 64 bits SPSS Statistics Version 27.0.1.0 SPSS and EXCEL. To determine the validity of the scale, exploratory and confirmatory analyses were performed. The exploratory factor analysis performed followed the five-step exploratory analysis protocol of [17]: (1) Suitability of data was assessed with the Kaiser–Meyer–Olkin measure and Bartlett’s sphericity test for measuring sampling adequacy, with a KMO ≥ 0.5 and significancy (*p* < 0.5) as criteria. (2) Factor extraction with the cumulative percentage of variance of 50–60% was used as a criterion [11]. (3) Selection of the rotational model was used, in this case, with a maximum likelihood method. (4) Run analysis was used. (5) Interpretation was used.

For the CFA, the mathematical algorithm used to identify estimates for each parameter was the maximum likelihood estimator (ML), following [11]. For assessing the model validity, model fit was examined using several goodness-of-fit indexes: CMIN/DG, the comparative goodness-of-fit index (CFI), the goodness-of-fit index (GFI), the Tucker–Lewis Index (TLI), and the Root Mean Squared Error of Approximation (RMSEA). Recommendations for evaluating model fit were followed from [18]. This analysis was carried out with the same sample as the EFA.

Reliability was calculated using Cronbach’s alpha and McDonald’s omega, and the criteria for its interpretation followed the recommendations of [11] of α ≥ 0.7 and ω ≥ 0.7. Regarding the distribution of items by subscale, a minimum saturation criterion of 0.40 was considered, following [19].

## 3. Results

### 3.1. Expert Validation Items Analysis

The intended sample of nine for the expert preliminary items analysis was sought. Thus, three PhDs in physical activity and sport sciences, four medical specialists (one in emergency medicine, one in cardiology, one in internal medicine, and one in sports medicine), and two with a master’s degree participated in this phase.

In Table 1, the assigned values from the nine judges for each of the items in the questionnaire are shown, along with the summation (∑xi), the maximum value (Mx), the content validity coefficient obtained for each item (CVCi), the probability of error for each item (Pei), and finally, the total content validity coefficient (CVCt). Additionally, the average values for each of the dimensions are collected.

A comparison was made of the obtained data applied using a formula for each item with the interpretative estimation scale [16]. In the end, the translated instrument, named “Prescription and Counseling of Physical Activity among Health Professionals in the Hospital Setting” (survey), has a validity and agreement index of 0.92 across the total items. Only two items have an average below 2.90 but above 0.85 (Table 2).

### 3.2. Exploratory Factor Analysis

An EFA was conducted on the nine items to assess the factor structure. Bartlett’s test of sphericity and the Kaiser–Mayer–Olkin measure of sample adequacy are presented in Table 3, both showing suitability for a factor analysis: a KMO value of 0.84, greater than [17], a recommendation of ≥0.5, and *p* < 0.5 in the Bartlett’s Test of sphericity were obtained (Table 3).

Following the criteria of item retention of 0.40 or more in [19], it was possible to extract two common factors related to the promotion of physical activity in patients with four items each (Table 4); they were named Advice on Physical Exercise and Application of Physical Exercise. The cumulative variance explained obtained was 52.4%.

### 3.3. Confirmatory Factor Analysis

The nine-item factor model was evaluated with the following adjustment indices: χ^2^ (23, N = 123) = 71.510, *p* < 0.001, CFI = 0.89, TLI = 0.83, GFI = 0.99, and RMSEA = 0.129. Following Hu and Bentler’s (1999) [18] criteria, the CMIN/DF, CFI, TLI, and RMSEA are unacceptable; the GFI is excellent.

### 3.4. Internal Consistency Reliability

The global Cronbach’s alpha in Table 5 of 0.85 (for nine items) indicates an adequate internal consistency [14].

The following table (Table 6) presents the reliability results for two factors of the instrument, calculated using McDonald’s omega (ω), a robust index that complements Cronbach’s alpha, especially useful when items do not meet the assumption of unequal variances.

**Factor 1: Advice on Physical Exercise**

This factor presented a value of ω = 0.852, which allows us to interpret the internal consistency as excellent according to [20].

**Factor 2: Application of Physical Exercise**

The reliability analysis of this factor revealed ω = 0.798, which can be interpreted as good internal consistency [20].

In Table 7, we present a detailed item-by-item analysis of the responses, highlighting key findings on the most common practices and the most neglected ones. This provides empirical evidence of the gaps between knowledge, perception, and action in exercise prescription. It also shows a proposal for their translation into English.

## 4. Discussion

Physical activity constitutes a cornerstone in the prevention and treatment of non- communicable chronic diseases (NCDs), as established by [1]. However, its effective implementation in daily clinical practice faces numerous challenges.

Countries like the Netherlands have initiated projects such as [6] that, as an objective, provide patients counselling about PA in order to prevent and control chronic diseases, improve recovering, and allow for healthy aging through the development of strategies of implementation of E = M; in Mexico, studies such as [21] have been conducted to determine the effects of an intervention of prescribing physical exercise for the development of functionality in patients after surgery. However, there is little evidence about the effects of a physical exercise prescription during clinical treatment or where synergy is performed among physicians, nutritionists, and exercise science professionals. In this article, a new contribution has allowed that the work be evaluated in this aspect within second-level attention.

### 4.1. Detailed Analysis of Factor 1: Advice on Physical Exercise

This factor consistently grouped items related to active communication between physicians and patients, highlighting a clear pattern in the responses. The high reliability of the construct (ω = 0.852), supported by significant factor loadings and elevated internal consistency, suggests that these items coherently measure a single underlying dimension: the quality of communication by the physician.

This finding aligns with recent research, such as the study by [22], which identifies physician–patient verbal interaction as a key predictor for adherence to physical activity. Nevertheless, there remains a concerning gap between discourse and clinical practice: only 44.9% of professionals assess PA through standardized physical tests. This discrepancy reveals a critical opportunity to strengthen the implementation of concrete tools that translate theoretical communication into tangible actions during consultations.

### 4.2. Detailed Analysis of Factor 2: Application of Physical Exercise

Despite the analyzed factor demonstrating acceptable reliability (ω = 0.798), its internal structure reveals certain aspects that warrant a critical review. Specifically, the low factor loading of the item related to the evaluation of physical activity through physical tests (0.460) suggests a weak integration with the underlying theoretical construct. Such discrepancies are not unusual in contexts where the application of standardized tools is affected by real clinical conditions. Recent research has indicated that the implementation of physical tests in clinical settings faces significant barriers, such as a lack of time, resources, or insufficient professional training in objective evaluation techniques [23]. In many cases, standardized physical tests require specific equipment, adequate spaces, and trained personnel, which are not always available in clinical settings, especially in primary care or centers with logistical constraints [24]. This situation can generate considerable variability in the way these assessments are implemented and, consequently, bias in the obtaining of results, which, in turn, affects their structural validity within broader or more specific measurement scales or models. Furthermore, it has been shown that health professionals’ perception of the applicability of these tests can influence their use, which introduces another level of variability [25]. Therefore, these findings not only highlight structural limitations in measuring the construct but also point to the need for strategic interventions aimed at improving the capacity of clinical environments to apply physical evaluations systematically and objectively. This may involve both methodological reformulation of the items and strengthening clinical competencies through continuous training and access to adequate resources [1]. In summary, although the factor manages to capture relevant dimensions, its heterogeneous operationalization opens an important pathway to refine its measurement and increase its applicability in other real-world scenarios.

### 4.3. Comparative Analysis Between Factors

The comparative study between the two factors reveals notable differences in their consistency and stability. Firstly, factor 1 demonstrated greater reliability (ω = 0.852) compared to factor 2 (ω = 0.798), indicating that its items measure the underlying construct with greater accuracy. However, both factors exhibit reliability above the acceptable level [19]. Factor 2 presented a wider confidence interval, suggesting greater variability in its measurements and, consequently, lower stability in its results.

Finally, it should be noted that all items showed adequate factor saturations [19], except for the item “Do you think it is necessary for exercise to be prescribed by specialized professionals to support the prevention or treatment of NCDs?”, indicating that all of this corresponds to an identified dimension.

This marked difference between verbal communication and documented communication is particularly relevant in light of research such as that of [26], who demonstrated that written guidance significantly improves long-term adherence to physical exercise in patients.

In the realm of clinical evaluation, the data present a revealing paradox: although 44.9% of professionals recognize the utility of standardized physical tests for evaluating physical activity, more than half (55.1%) rarely or never provide documented instructions. This apparent contradiction reflects, as noted by [27], the structural limitations faced by health systems, including time constraints, insufficient resources, and gaps in specialized training.

The situation revealed is very concerning when analyzing referral processes. There is overwhelming consensus (85%) on the necessity for specialists in physical activity to prescribe exercise programs; however, in actual clinical practice, nearly half of the physicians (48.8%) almost never make these referrals. These limitations, indirectly highlighted in the call for a ‘multisectoral approach’ in the WHO’s Global Action [28], reflect challenges both globally and locally, where theory does not always translate into practice due to failings in those who manage health systems as well as those who operate within them.

### 4.4. Detailed Clinical Implications

The findings of this study present important challenges and opportunities for optimizing clinical practice in promoting physical exercise.

First, improving evaluation processes requires the systematic implementation of validated and quickly applicable tools in clinical contexts. Recent research highlights the usefulness of standardized questionnaires such as the International Physical Activity Questionnaire (IPAQ) and the Physical Activity Readiness Questionnaire (PAR-Q) [29], as well as brief functional tests like the sit-to-stand test [30], which allow for objective assessment without consuming valuable consultation time.

Second, optimizing physician–patient communication demands multifaceted interventions. Recent studies emphasize that standardized tools, such as digital forms for exercise prescription or infographics tailored to different literacy levels, significantly improve the clarity and follow-up of recommendations [31,32]. Additionally, integrating practical workshops on empathetic communication and establishing collaborative goals has been shown to increase adherence even in reluctant patients, addressing not only educational barriers but also psychosocial ones [33].

Finally, strengthening referral systems requires coordinated and concrete institutional actions. The creation of standardized protocols, such as those proposed by [34], along with established shared responsibility agreements between interdisciplinary networks that include physicians, physical therapists, and exercise science specialists, could resolve the current paradox between theoretical assessment and actual practice for patient referral.

In summary, the effective transformation of these clinical practices will require the following: first, the adoption of brief but scientific assessment instruments; second, the implementation of standardized communication resources; and third, the institutionalization of evidence-based referral systems. As the guidelines in [1] emphasize, only through this multidimensional approach can the gap between current knowledge and its application in daily patient care be overcome.

### 4.5. Evidence-Based Intervention Proposals

The findings of this study suggest the need to implement multilevel interventions to improve the promotion of exercise prescription in clinical settings. At the individual level, it is recommended to conduct practical workshops aimed at healthcare professionals, focusing on three key competencies: first, rapid evaluation of physical exercise using validated tools (for example, the Rapid Assessment of Physical Activity (RAPA) questionnaire adapted to clinical contexts [35]); second, effective communication techniques based on motivational interviewing enhanced with self-determination strategies [36]; and third, basic principles of exercise prescription adapted to different populations, including approaches for older adults and chronic patients [37].

At the institutional level, interventions should aim to create support systems for clinical practice. This includes the development of standardized flowcharts for referring patients, clinical record systems that include metrics for physical exercise, and strategic partnerships with community exercise centers [34]. As suggested by the evidence from [38], the disconnection between scientific research and practice in motor competence and physical fitness reflects multisystemic barriers. The creation of interdisciplinary networks could overcome these limitations, ensuring more effective patient follow-up.

In terms of public policy, it is proposed to systematically integrate physical exercise into national clinical guidelines for various chronic conditions, following the model of the WHO guidelines [29]. Concurrently, the creation of professional incentives (both financial and for curriculum development) for those who demonstrate adherence to these protocols, along with funding for accessible community programs, could bridge the gap between medical recommendations and actual access to exercise programs [39].

These interventions, implemented in a coordinated manner, could transform the current fragmented approach into a comprehensive system where the prescription of physical activity is as systematic as any other medical treatment. As highlighted by international recommendations, this multilevel approach is essential for achieving significant impacts on population health [1].

### 4.6. Instrument Reliability and Construct Validity

The instrument used demonstrated some of the highest psychometric properties, evidenced by a Cronbach’s alpha value of 0.857, along with a McDonald’s omega value of 0.852 for factor 1 (Communication–Counseling) and for factor 2 (Referral–Evaluation). These values significantly exceed the thresholds recommended by [40], confirming the reliability of the questionnaire for evaluating these dimensions.

### 4.7. Limitations and Future Research

While this study provides valuable evidence regarding clinical practices related to the promotion of physical activity in a secondary-level hospital, it is important to recognize certain methodological limitations that could affect the generalization of the obtained results. First, the research was conducted with a sample confined to a specific geographic and sociocultural context, which limits the possibility of extrapolating the findings to other environments [41]. Therefore, the authors recommend the usage of this instrument to evaluate the context of exercise prescription practice among physicians through different healthcare levels, including primary, secondary, and tertiary care settings.

This aspect aligns with the literature highlighting how beliefs, cultural norms, and socioeconomic conditions—such as those related to gender roles, religion, or access to public spaces—can vary significantly between communities, impacting the generalization of physical activity interventions [41]. Additionally, the data collected through questionnaires could be subject to social desirability bias, where participants may have provided responses they deemed more acceptable rather than faithfully reflecting their actual clinical practice [42]. A third important limitation lies in the reliance on self-reports instead of direct observations or objective measurements, which could affect the validity of the collected data [43].

These limitations open up important opportunities for future research in this field. It would be particularly valuable to develop longitudinal studies that assess the medium- and long-term impact of different interventions designed to improve exercise prescription in clinical settings. Additionally, qualitative research, like that of [44], provides a deep understanding of the perceived barriers faced by healthcare professionals in promoting physical activity after gestational diabetes, highlighting both institutional factors (e.g., lack of continuity in postnatal care) and personal beliefs (perceptions of inconvenience or lack of training). These findings further emphasize the need for specific interventions that address not only the structural limitations of the healthcare system but also the attitudes and skills of health personnel to facilitate effective conversations about healthy lifestyles.

Finally, in recent years, it has been recognized that healthcare systems are implementing the use of technology. Therefore, it is a priority to evaluate the potential and use of digital tools (such as mobile apps or telemedicine platforms) to overcome some of the limitations identified in this study. For example, recent systematic reviews like that of [45] demonstrate that digital interventions such as mobile apps, wearables, and smart messaging are effective in significantly increasing daily step counts in university populations (SMD = 0.64; *p* < 0.001), a key indicator of physical activity. However, the same study reveals that these tools still face challenges in impacting higher-intensity activities or reducing sedentary behavior, suggesting the need to complement them with personalized strategies or human support. These findings reinforce the importance of integrating technological solutions with a design centered on the specific gaps identified.

## 5. Conclusions

The instrument used for this exploratory study helped to determine the dual perspective of promoting prescribed physical exercise among participating physicians, such as identifying that there is indeed effective verbal communication during consultations, where professionals convey recommendations on PA in a clear and motivating manner. However, the instrument helped to contrast that there are critical gaps in the formal evaluation of the levels of FA of the patients as well as a notable discrepancy between the theoretical assessment of the importance of PA and its translation into specific derivations to specialized physical exercise programs.

## Figures and Tables

**Table 1:** Assigned values from the 9 judges.

Items	Judges									Formulas				
	Judges 1	Judges 2	Judges 3	Judges 4	Judges 5	Judges 6	Judges 7	Judges 8	Judges 9	Sx1	Mx	CVCi	Pei	CVCtc
item 1	9	11	11	12	12	12	7	12	8	94	7.83333333	0.87037037	2.58117 × 10^−9^	0.87037037
item 2	12	8	10	11	11	12	9	12	12	97	8.08333333	0.89814815	2.58117 × 10^−9^	0.89814815
item 3	10	9	12	9	12	12	11	12	12	99	8.25	0.91666667	2.58117 × 10^−9^	0.91666666
item 4	12	12	10	12	9	12	11	12	12	102	8.5	0.94444444	2.58117 × 10^−9^	0.94444444
item 5	12	7	12	9	12	12	11	12	12	99	8.25	0.91666667	2.58117 × 10^−9^	0.91666666
item 6	10	12	12	12	12	12	11	12	10	103	8.58333333	0.9537037	2.58117 × 10^−9^	0.9537037
item 7	12	12	12	12	12	12	11	11	10	104	8.66666667	0.96296296	2.58117 × 10^−9^	0.96296296
item 8	12	12	9	12	12	12	6	12	12	99	8.25	0.91666667	2.58117 × 10^−9^	0.91666666
item9	11	12	12	11	10	12	11	12	9	100	8.33333333	0.92592593	2.58117 × 10^−9^	0.92592592
													Total sum of the CVCtc	8.30555553
													General average	0.9228395

**Table 2:** Estimated scale for interpreting content validity coefficient (Pedrosa et al., 2014 [16]).

(a) Less than 0.60: Unacceptable validity and agreement.
(b) Equal to or greater than 0.60 and less than or equal to 0.70: Deficient validity and agreement.
(c) Greater than 0.71 and less than or equal to 0.80: Acceptable validity and agreement.
(d) Greater than 0.80 and less than 0.90: Good validity and agreement.
(e) Greater than 0.90: Excellent validity and agreement.

**Table 3:** Exploratory Factor Analysis.

KMO and Bartlett’s Test		
Kaiser–Meyer–Olkin Measure of Sampling Adequacy		0.844
Bartlett’s Test of Sphericity	Approx. chi-square	480.107
	gl	36
	Sig.	0.001

**Table 4:** Factor loadings of clinical practices related to the prescription and evaluation of physical exercise.

	Factor 1	Factor 2
Do you advise physical exercise (verbal or written) to prevent non-communicable diseases?	0.820	
Do you advise physical exercise (verbal or written) to treat non-communicable diseases?	0.784	
Do you talk about physical exercise with your patients?	0.702	
How often do you ask your patients about their PA level?	0.606	
Do you think it is necessary to prescribe exercise by specialized professionals to support the prevention and treatment of CNDs?		
Do you provide written instructions about a physical exercise program to your patients?		0.799
How often do you refer patients to specialized personnel for a fitness assessment?		0.695
Do you provide verbal instructions on any physical exercise program to your patients?		0.677
Do you consider it important to evaluate PA through a physical test as part of your clinical exam?		0.460

The table presents the structure of two main factors derived from exploratory factor analysis, which grouping the clinical practices related to the promotion of physical activity in patients; loadings below 0.40 are not presented.

**Table 5:** Reliability statistics.

Cronbach’s Alpha	Elements
0.85	9

**Table 6:** Reliability by factors.

Factor 1 Advice on Physical Exercise	McDonald’s ω
Estimated	0.852
95% CI lower limit	0.810
95% CI upper limit	0.894
**Factor 2 Application of Physical Exercise**	
Estimated	0.798
95% CI lower limit	0.742
95% CI upper limit	0.853

**Table 7:** Findings by item.

Items	Percentages	Description
(1) How often do you ask your patients about their PA level?	63.8% frequently or very frequently. Only 4.7% never.	Finding: Most physicians actively inquire about physical activity.
(2) Do you consider it important to evaluate PA through a physical test as part of your clinical exam?	44.9% frequently or more.	Almost half of the physicians do not prioritize formal assessments of physical activity.
(3) How often do you refer patients to specialized personnel for a fitness assessment?	48.8% never or very rarely. Only 26.8% frequently or more.	Low integration with specialized physical exercise professionals.
(4) Do you provide verbal instructions on any physical exercise program to your patients?	60.6% provide verbal instructions at least “occasionally,” combining all the other options: occasionally, frequently, and very frequently. 29.1% perform this never or very rarely.	Although most physicians provide verbal instructions, about 30% of them rarely provide them, suggesting that these opportunities should be utilized to improve active communication.
(5) Do you provide written instructions about a physical exercise program to your patients?	55.1% never or very rarely. Contrast: 60.6% give verbal instructions occasionally or more.	Oral communication predominates over written communication.
(6) Do you advise physical exercise (verbal or written) to prevent non-communicable diseases?	70.9% perform this frequently or very frequently (38.6% + 32.3%). Only 3.1% state they never advise it, and another 9.4% advise it very rarely.	Most physicians recommend physical exercise as a strategy for the prevention of non-communicable diseases very frequently.
(7) Do you advise physical exercise (verbal or written) to treat non-communicable diseases?	61.1% advise it frequently or very frequently (34.6% + 31.5%) The percentage of those who never advise it rises to 7.1%, compared to 3.1% in prevention.	Although recommendations also predominate, there is a slight decrease in the frequency with which physicians prescribe exercise as treatment.
(8) Do you talk about physical exercise with your patients?	60.6% frequently or very frequently. Only 0.8% never	Verbal communication is a very common practice between the physician and the patient.
(9) Do you think it is necessary to prescribe exercise by specialized professionals to support the prevention and treatment of CNDs?	85% of physicians consider it necessary or very necessary. Frequently–very frequently: 37.8% + 47.2%. Only 4.7% consider it very rarely necessary.	There is a high recognition of the importance of the role of exercise specialists, but this contrasts with the low real frequency; they never or very rarely make referrals.

## Data Availability

The dataset used and analyzed in this study is available from the corresponding author. The data are not publicly available. Regarding the visibility of the data presented in the article, we believe that, should any questions arise from readers, it would be preferable for them to contact the corresponding author directly. This approach would help minimize potential misinterpretations and ensure an accurate and informed response.
